# Pelvic Kidney With Contralateral Renal Vascular Variants: A Rare Anatomical Case From Djibouti

**DOI:** 10.7759/cureus.108258

**Published:** 2026-05-04

**Authors:** Rahma Abdillahi, Saredo Aganeh, Wassim Guermazi, Amarkak Abdillahi, Ali Ghorbel

**Affiliations:** 1 Biology Department, Faculty of Sciences of Sfax, University of Sfax, Sfax, TUN; 2 Radiology Department, Djibouti National Police Hospital, Djibouti Ville, DJI; 3 Radiology Department, Djibouti Military Hospital, Djibouti Ville, DJI; 4 Anatomy Laboratory, Faculty of Health of Sfax, University of Sfax, Sfax, TUN

**Keywords:** case report, ct urography, pelvic kidney, renal ectopia, vascular variations

## Abstract

A pelvic kidney is a rare congenital renal ectopia frequently associated with vascular anomalies. Knowledge of these variations is essential for diagnostic evaluation and surgical planning. We report the case of a 35-year-old man in whom a right pelvic kidney with unusual vascular variations was detected on computed tomography urography. A single renal artery arising from the anterior aspect of the aortic bifurcation supplied the ectopic kidney, which was drained by three right renal veins. In addition, the contralateral kidney, located in its normal anatomical position, also exhibited vascular variations, including two left renal arteries and two left renal veins. Pelvic kidneys are often associated with complex vascular patterns, as demonstrated here. Therefore, knowledge of these anatomical variations is crucial for accurate diagnosis and for safe surgical or interventional procedures, particularly in the management of renal vascular diseases and in the context of kidney transplantation.

## Introduction

A pelvic kidney is defined as a kidney located below the horizontal plane of the iliac crest. This congenital anomaly results from incomplete or abnormal ascent of the metanephros during embryological development, leading to its persistence within the pelvic cavity [1]. The reported incidence of pelvic kidneys is approximately 1 in 1,000 births, although many cases remain asymptomatic and are discovered incidentally [1].

Renal ectopia may occur on either side, with a slight right-sided predominance according to several anatomical and radiological series. The ectopic kidney is most commonly situated in the presacral region and is frequently associated with complex vascular anatomy, characterized by significant variations of the renal pedicle, including multiple or aberrant renal arteries as well as anomalies of venous drainage [2,3]. Several studies have emphasized that these vascular variations result from the persistence of transient embryonic arteries, thereby explaining the diversity of vascular patterns observed.

A comprehensive understanding of renal ectopia and its vascular particularities is of critical importance for urologists, radiologists, vascular surgeons, and transplant specialists. Such knowledge is especially essential in the context of open or laparoscopic renal surgery, living donor nephrectomy, endovascular procedures, and renal transplantation, where accurate preoperative identification can significantly reduce the risk of iatrogenic complications, including vascular injury and intraoperative technical difficulties [4].

In this context, we report a case of a right pelvic kidney with unusual vascular variations, incidentally identified during contrast-enhanced computed tomography urography (CT urography). Furthermore, we provide a review of the literature addressing the spectrum of vascular configurations associated with pelvic kidneys and their reported frequencies, in order to better contextualize this case within current anatomical and clinical knowledge.

## Case presentation

A 35-year-old man with no significant medical, surgical, or family history presented with right-sided lower back pain. He was alert and hemodynamically stable. Physical examination revealed a palpable, well-defined mass in the right iliac fossa.

**Figure 1:**
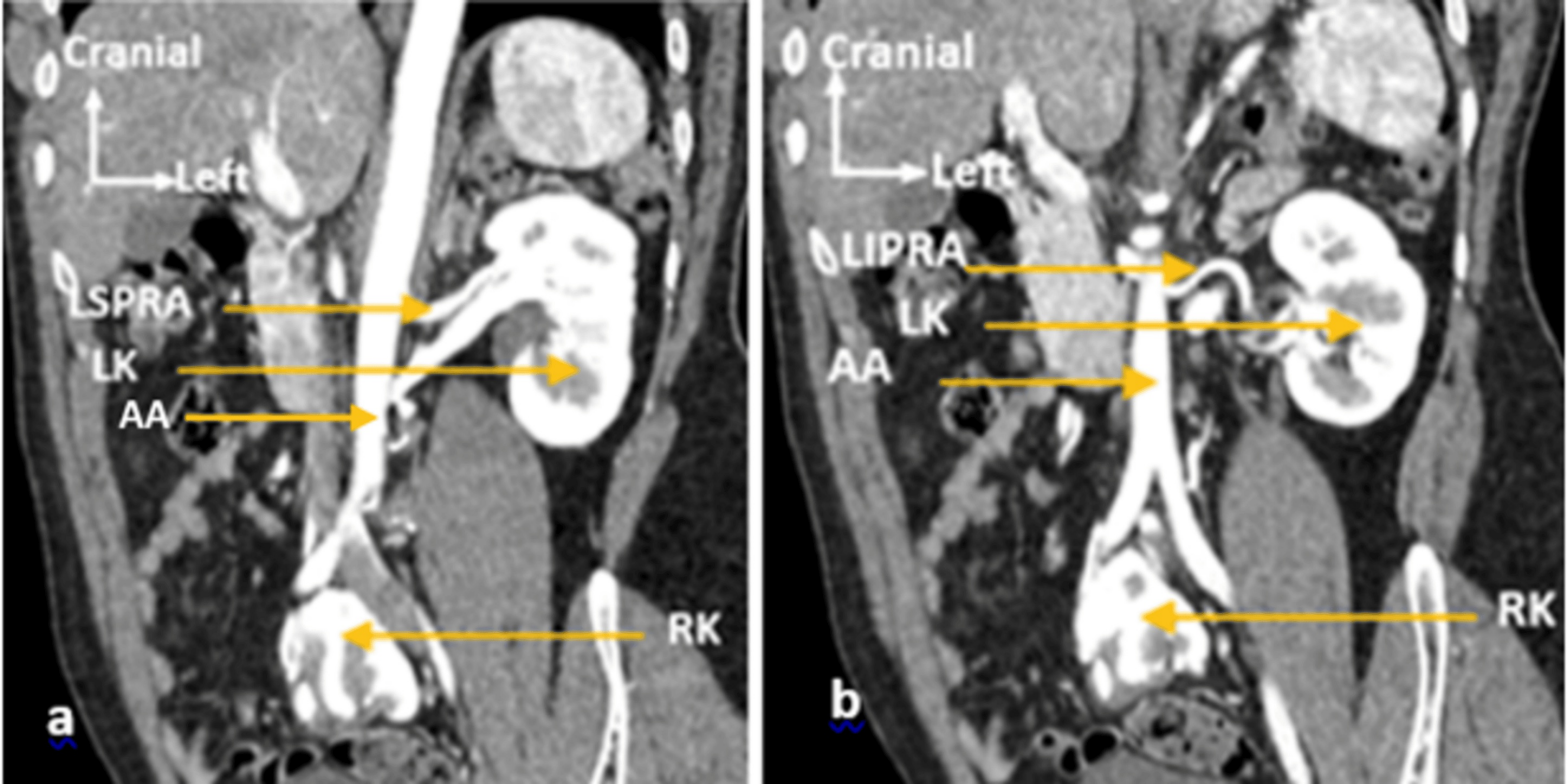
Frontal 2D CT section showing (a) the left superior polar renal artery (LSPRA) and (b) the left inferior polar renal artery (LIPRA). LK: Left kidney; AA: abdominal aorta; RK: right kidney

**Figure 2:**
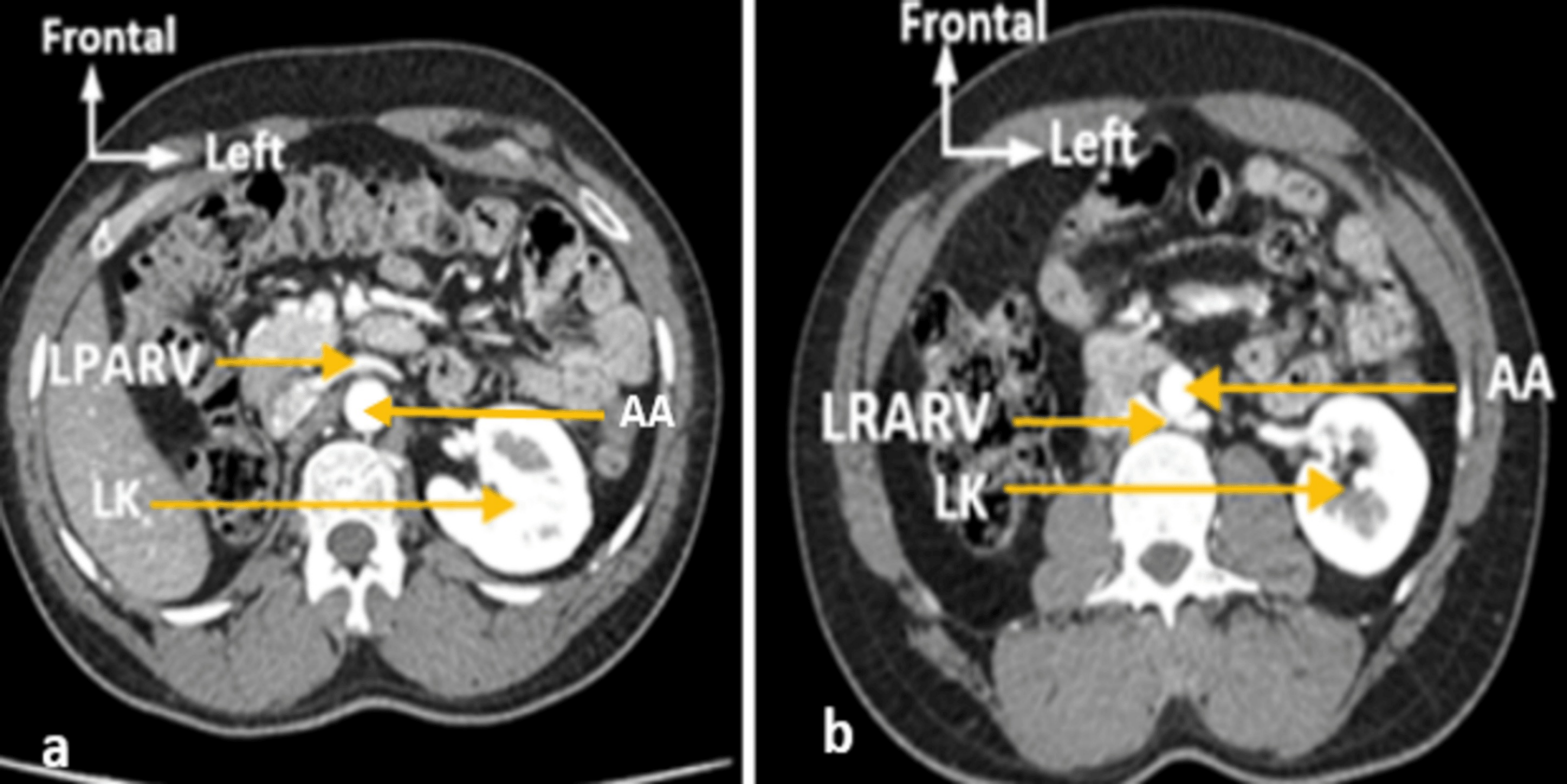
Axial 2D CT section showing (a) Left pre-aortic renal vein (LPARV) and (b) Left retro-aortic renal vein (LRARV). AA: Abdominal aorta; LK: left kidney

Abdominopelvic CT showed a normally positioned left kidney of normal size with vascular variations, including two polar arteries from the abdominal aorta and pre-aortic and retro-aortic veins draining into the inferior vena cava (Figures 1, 2).

**Figure 3:**
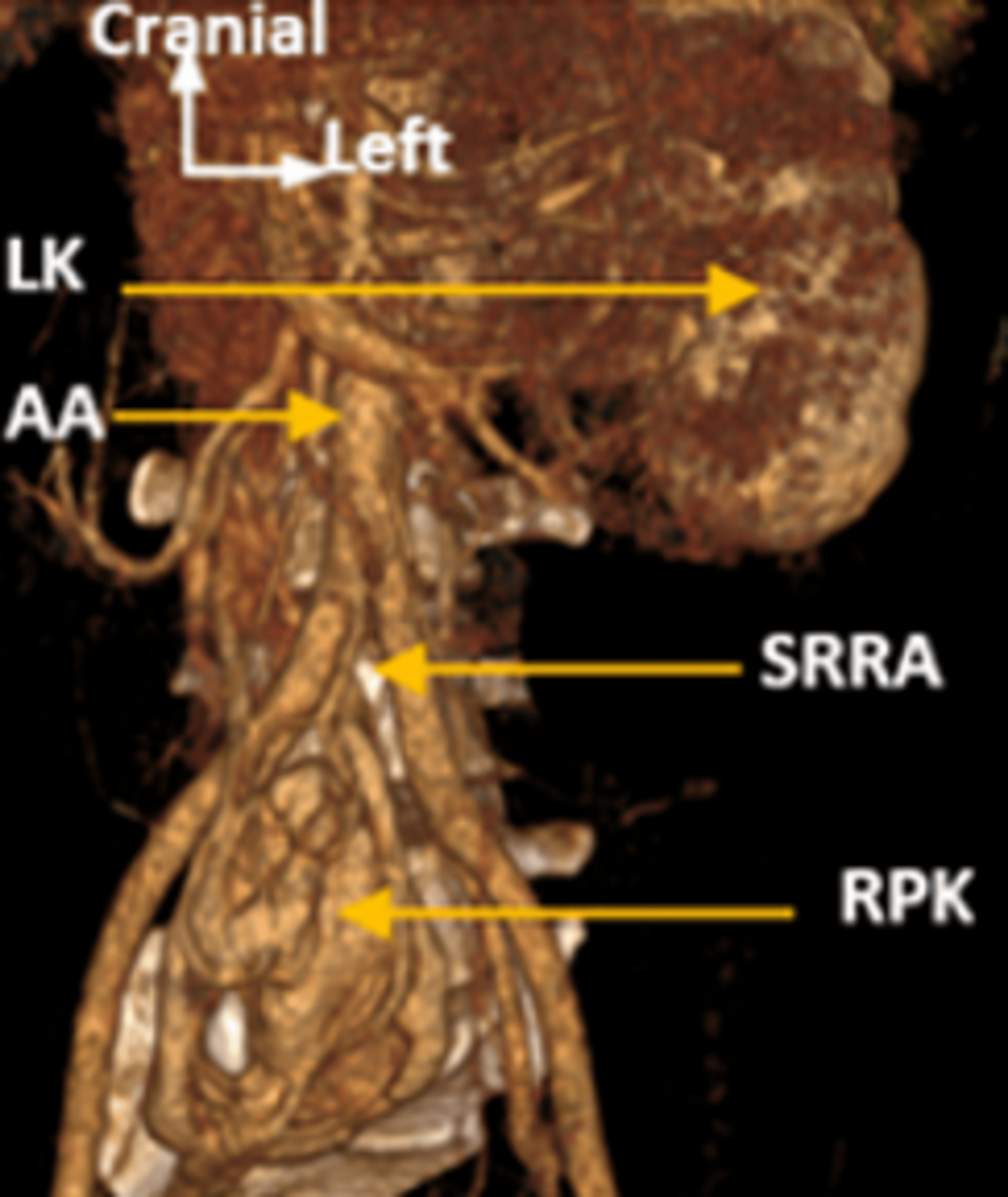
Oblique frontal CT section with 3D reconstruction showing the single right renal artery (SRRA). RPK: Right pelvic kidney; AA: abdominal aorta; LK: left kidney

A right pelvic kidney measuring 10 cm in length was identified, containing a 12-mm calcified renal stone (1276 HU) and exhibiting atypical vascular anatomy. Arterial supply consisted of a single renal artery originating from the anterior aortic bifurcation and coursing anterior to the inferior vena cava to the hilum (Figure 3).

**Figure 4:**
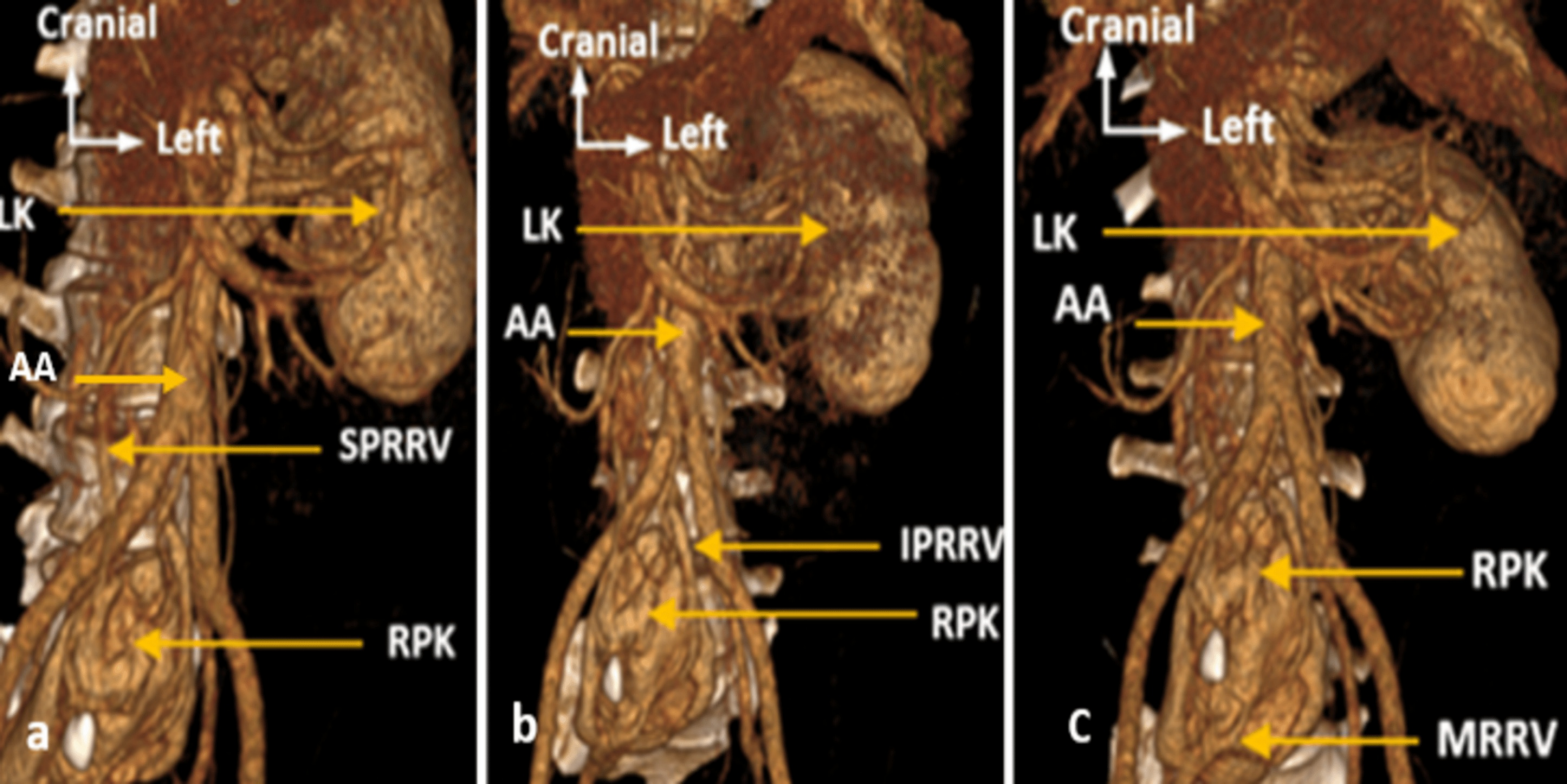
Oblique frontal CT section with 3D reconstruction showing (a) Superior polar right renal vein (SPRRV), (b) Inferior polar right renal vein (IPRRV), and (c) Middle right renal vein (MRRV). AA: Abdominal aorta; RPK: right pelvic kidney; LK: left kidney

Venous drainage included three veins: an inferior vein draining into the confluence of the inferior vena cava, a superior vein draining directly into the inferior vena cava, and a medial vein draining into the right internal iliac vein (Figures 4a-4c).

The diagnosis of a right pelvic kidney with atypical vascular anatomy was made. Although structurally normal, pelvic kidneys can occasionally cause non-specific abdominal or lumbar pain, sometimes with digestive or urinary symptoms. In this case, lumbar pain prompted CT imaging, incidentally revealing the ectopia. CT urography is essential for confirming the diagnosis and assessing associated anomalies.

As the pelvic kidney had normal morphology and function, no surgical intervention was required. The patient was reassured that the condition represented an anatomical variation rather than a pathological disorder, and clinical monitoring was recommended.

## Discussion

Embryologically, the intermediate mesoderm differentiates to form the definitive kidney, or metanephros, beginning around the eighth week of gestation. The metanephros initially develops in the pelvis and gradually ascends to the lumbar region. During this migration, the renal hilum, initially facing anteriorly, rotates medially approximately 90°, allowing the hilum and its vessels to attain their final anatomical orientation observed in adults. The kidneys remain retroperitoneal throughout this process. As they ascend, they cross key vascular structures, including the bifurcation of the primitive iliac arteries. Any disruption of this ascent or rotation may lead to positional anomalies, such as renal ectopia, with the kidney remaining near the iliac vessels [5].

Abnormalities of renal migration are frequently associated with variations in vascular anatomy. Multiple renal arteries are observed in nearly half of cases, a prevalence exceeding the estimated 30% reported in normally positioned kidneys [6]. When a single renal artery is present in ectopic kidneys, it most commonly originates from the aortic bifurcation [6]. In the present case, the ectopic kidney was supplied by a single right renal artery with a course anterior to the inferior vena cava terminating at the renal hilum. Clinically, renal ectopia is typically asymptomatic and is often detected incidentally during imaging studies [7].

The venous drainage of pelvic kidneys is highly variable and not fully characterized. In most cases, the renal veins are multiple, of small caliber, and drain into the inferior vena cava as well as the ipsilateral common iliac vein [6,8]. In our observation, the ectopic kidney demonstrated complex venous drainage, with three renal veins draining into distinct venous structures, including the inferior vena cava and the right internal iliac vein. This configuration highlights the anatomical complexity of ectopic kidneys and the potential technical challenges encountered during catheterization or surgical interventions.

A notable feature in this case is the presence of a vascular variation in the contralateral kidney despite its normal anatomical position [9]. The left kidney, although located between T12 and L3, demonstrated dual arterial supply from the abdominal aorta at different vertebral levels. Rare configurations of this type have been reported in the literature, including cases where the left kidney had three distinct renal arteries, emphasizing the anatomical variability of renal vasculature even in kidneys with normal positioning [10]. Knowledge of such arterial anomalies is crucial to minimize the risk of inadvertent injury to accessory arteries during renal surgeries, particularly in the context of kidney transplantation.

The venous drainage of the left kidney was atypical, consisting of both preaortic and retroaortic components. These variations arise from the complex embryological development of the venous system between the fifth and seventh weeks of gestation. During this period, the subcardinal and supracardinal veins form a network of anastomoses around the aorta. Persistence of certain channels can result in a circumaortic vein, in which anterior and posterior venous segments encircle the aorta before draining into the inferior vena cava. While well documented, such variations remain relatively rare in the anatomical and radiological literature [11,12].

Given the variability in number, origin, course, and drainage of renal vessels in ectopic kidneys, a thorough understanding of these anatomical relationships is crucial. This is particularly important in abdominal and pelvic surgery, where unrecognized vascular variations can result in significant intraoperative complications, including hemorrhage. Preoperative imaging, particularly contrast-enhanced CT angiography, is indispensable for accurately mapping vascular anatomy and guiding safe surgical planning.

This case report has several limitations. First, it describes a single patient, which limits the generalizability of the findings. Second, the observed vascular variations may represent individual anatomical peculiarities rather than common patterns. Third, the lack of long-term clinical follow-up and surgical correlation may restrict the broader clinical applicability of these observations.

## Conclusions

A pelvic kidney is a rare congenital anomaly often associated with complex and highly variable vascular patterns. Accurate recognition of these vascular variations is essential for radiologists and surgeons to reduce the risk of vascular injury during abdominal and pelvic interventions. In particular, inadequate preoperative identification of renal vascular anatomy may lead to major complications, including vascular misidentification, iatrogenic vascular injury, and significant hemorrhage during renal transplantation or endovascular procedures. Contrast-enhanced CT urography plays a crucial role in preoperative assessment by precisely delineating the vascular anatomy, thereby improving surgical planning and ensuring safer and more effective clinical management.
